# The preoperative cardiology consultation: indications and risk modification

**DOI:** 10.1007/s12471-017-1004-1

**Published:** 2017-05-31

**Authors:** M. W. Groot, A. Spronk, S. E. Hoeks, R. J. Stolker, F. van Lier

**Affiliations:** Department of Anesthesiology, Erasmus University Medical Center, Rotterdam, The Netherlands

**Keywords:** Preoperative Care, Referral and Consultation, Risk assessment

## Abstract

**Background:**

The cardiologist is regularly consulted preoperatively by anaesthesiologists. However, insights into the efficiency and usefulness of these consultations are unclear.

**Methods:**

This is a retrospective study of 24,174 preoperatively screened patients ≥18 years scheduled for elective non-cardiac surgery, which resulted in 273 (1%) referrals to the cardiologist for further preoperative evaluation. Medical charts were reviewed for patient characteristics, main reason for referring, requested diagnostic tests, interventions, adjustment in medical therapy, 30-day mortality and major adverse cardiac events.

**Results:**

The most common reason for consultation was the evaluation of a cardiac murmur (95 patients, 35%). In 167 (61%) patients, no change in therapy was initiated by the cardiologist. Six consultations (2%) led to invasive interventions (electrical cardioversion, percutaneous coronary intervention or coronary artery bypass surgery). On average, consultation delayed clearance for surgery by two weeks.

**Conclusion:**

In most patients referred to the cardiologist after being screened at an outpatient anaesthesiology clinic, echocardiography is performed for ruling out specific conditions and to be sure that no further improvement can be made in the patient’s health. In the majority, no change in therapy was initiated by the cardiologist. A more careful consideration about the potential benefits of consulting must be made for every patient.

## Introduction

Since the introduction of our speciality, anaesthesiologists have been at the forefront of attempts to improve patient safety [1]. For preoperative risk evaluation and modification, preoperative assessment is widely introduced to obtain information about the patient’s state of health and to collect data on previous treatments [2, 3]. The ultimate goals of preoperative assessment are to evaluate perioperative risk, optimise patient status and to reduce the patient’s surgical and anaesthetic morbidity or mortality [4, 5].

During preoperative assessment, consultation is common and might modify the outcome if further information leads to a different anaesthetic regime or if interventions are performed based on further assessment. If no change in management is needed it can lead to unnecessary and potentially harmful investigations [6] and furthermore may delay surgery. As 42% of the overall complications in non-cardiac surgery are caused by cardiac complications, the cardiologist is still the most frequently consulted specialist in the preoperative work-up [7, 8]. In our clinical practice the cardiologist is the preferred specialist for the treatment of all cardiovascular diseases, including hypertension and hypercholesterolaemia. The relevance of these consultations has been disputed. While usefulness is described by Kleinman [9], others report an overuse of preoperative cardiac consultation and claim that these consultations gave little advice that truly impacts management [10–13]. Unfortunately, most of these studies have significant limitations, as they were performed before the introduction of widely accepted guidelines [14–18], before the implementation of preoperative consultation by the anaesthesiologist in an outpatient clinic and investigated patients were often referred by the surgeons themselves*.*

Despite earlier statements in the literature about overuse of preoperative cardiac consultation, patients are still being referred to the cardiologist as part of their preoperative work-up. However, it is unclear what the impact of these guidelines is on the referral rate and whether these guidelines are strictly followed for preoperative optimisation. Secondly, it is unknown what the delay in surgery is due to preoperative consultation. The purpose of this study is to gain further insight into both these issues during the preoperative period.

## Methods

This is a retrospective study of all consecutive patients referred to a cardiologist after preoperative screening in the outpatient anaesthesiology clinic of the Erasmus University Medical Centre, Rotterdam, the Netherlands between November 2011 and January 2014. No ethical approval was required because the study is based on anonymised retrospective data. As per hospital protocol, all patients ≥18 years scheduled for elective non-cardiac surgery are screened by an anaesthesiologist, an anaesthesiologist-in-training or a trained physician assistant. The European Society for Cardiology Guidelines for preoperative assessment were used for the preoperative work-up in our hospital within the specified time frame [14–18]. During the inclusion period, a total of 24,174 patients were preoperatively screened, 278 of whom were referred to the cardiology department and included in this study. Patients were excluded if the referral was unrelated to the planned operation, these consults were requested by patients themselves for other reasons and not on medical grounds. Baseline characteristics were obtained and the Revised Cardiac Risk Index [19] was calculated. The main reason for referral was noted and classified into nine different categories: possibility of valve abnormality, angina pectoris (stable and unstable), ECG changes, atrial fibrillation (new-onset and known disease), dyspnoea, hypertension, anticoagulation management, general evaluation, and other. Only if the anaesthesiologist initiating the consult asked for a ‘general evaluation’, were referrals marked as such.

Medical charts were reviewed for patient characteristics (i. e. age, gender and ethnic background), 30-day postoperative all-cause mortality and major adverse cardiac events (MACE), diagnostic tests requested by the cardiologist (i. e. ECG, transthoracic echocardiography, exercise stress ECG, myocardial perfusion scan, Holter monitoring, stress echocardiogram, coronary angiogram, CT coronary angiogram, transoesophageal echocardiogram, CT aorta and Brugada syndrome test), invasive interventions (i. e. electrical cardioversion, percutaneous coronary intervention and coronary artery bypass surgery) and adjustment in medical therapy (i. e. start or change of dosage of a cardiac drug).

Median, including interquartile range (IQR), was used for variables not normally distributed. Normal distribution of the data was determined both visually using histograms and Q‑Q plots and with the Shapiro-Wilk test. Categorical data are presented as absolute values and percentages. All calculations were performed using IBM SPSS 22.0 statistical software (SPSS Inc., Chicago, Illinois, USA).

## Results

**Fig. 1:**
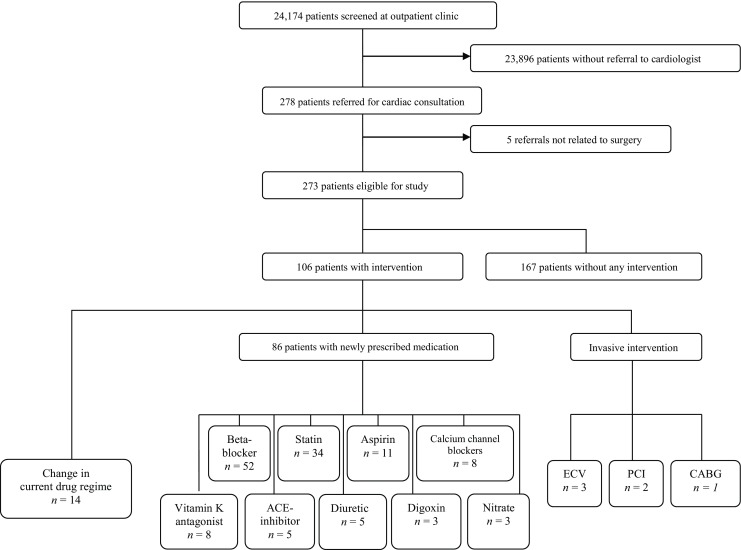
Flowchart; *ECV* electrical cardioversion, *PCI* percutaneous coronary intervention, *CABG* coronary artery bypass surgery

The medical records of 24,174 patients were screened. A total of 278 (1%) were referred for cardiac consultation, 5 of whom were excluded (Fig. 1), resulting in a study population of 273 patients. The median age was 65 (IQR 56–74), with a range of 18 to 97 years; 54% were male and 82% Caucasian. Thirty-day mortality rate was 1.1% (*n* = 3) and MACE occurred in 2.6% (*n* = 6): congestive heart failure in 3 patients, myocardial infarction in 2 patients, and cardiovascular death in 1 patient.

**Fig. 2:**
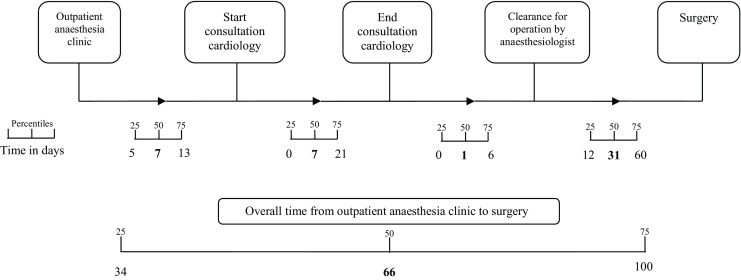
Waiting time preoperative term, per section

The most common reason (*n* = 95, 35%) for consultation was related to the possibility of valve pathology; either a new murmur was heard during physical examination or evaluation of a previously known valve abnormality was requested. Other preoperative issues leading to consultation of a cardiologist are listed in Table 1. The median time between visiting the outpatient anaesthesiology clinic and starting cardiac consultation was 7 days (IQR 5–13). Median duration was an additional 7 days (IQR 0–21) (Fig. 2).

**Table 1:** Main reason for referring a patient to the cardiologist (*n* = 278)

	Number of patients (%)
Evaluation of valve abnormality	95 (35%)
General evaluation^a^	44 (16%)
Angina pectoris^b^	42 (15%)
ECG changes	24 (9%)
Atrial fibrillation^c^	21 (8%)
Dyspnoea	18 (7%)
Hypertension	6 (2%)
Anticoagulation management	5 (2%)
Other	18 (7%)
*Total*	*273 (100%)*

^a^Actual request by initiator of consultation

^b^Stable and unstable

^c^New onset and known disease

In the majority of the patients (214, 87%) the cardiologist ordered more than one diagnostic test. ECG was performed most frequently (269 patients, 99%), followed by transthoracic echocardiography in 192 cases (72%). Other performed tests were: exercise stress ECG (*n* = 29, 11%), myocardial perfusion scan (*n* = 18, 7%), Holter monitoring (*n* = 13, 5%), stress echocardiogram (*n* = 9, 3%), coronary angiogram (*n* = 6, 2%), CT coronary angiogram (*n* = 3, 1%), transoesophageal echocardiogram (*n* = 1, 0.4%), CT aorta (*n* = 1, 0.4%) and Brugada syndrome test (*n* = 1, 0.4%).

In 167 cases (61%), no change in therapy was initiated by the cardiologist (Fig. 1). Three patients (1%) underwent electrical cardioversion, all because of new-onset atrial fibrillation found during preoperative assessment. Percutaneous coronary intervention was performed in two patients (<1%) preceding non-cardiac surgery. These patients were primarily referred because of ‘angina pectoris’ and ‘a change in ECG’, respectively. One patient had coronary artery bypass surgery. In 14 patients (5%) doses of their current drug regime were changed. New medication (with or without a change in current prescriptions) was started in 86 patients (32%). The most frequently prescribed new drug was a β-blocker, started in 52 patients (19%). In 17 out of 52 patients (33%), these were started because of guideline recommendation, followed by (new-onset) atrium fibrillation (25%), the presence of coronary artery disease (21%) and treatment for hypertension (15%). The majority of patients (96%) in whom β‑blockers were started were at a low risk for adverse cardiac events, as reflected by a revised Cardiac Risk Index of ≤1 [19].

## Discussion

In this study we analysed 273 referrals to the cardiologist for preoperative evaluation. We evaluated the diagnostic tests and changes in therapy initiated by the cardiologist. In the majority of patients in our study population (61%) there was no change in therapy after cardiac consultation. In 2% consultation led to invasive interventions (electrical cardioversion, percutaneous coronary intervention or coronary artery bypass surgery) and in 37% of the cases pharmacological changes were made, either a change in the current drug regime or a new medication was prescribed.

Secondly, we found that the process of cardiac consultation until clearance for surgery takes approximately two weeks. It is unclear whether this time delays the planned date of surgery, since a date for the operation could have been planned well ahead due to waiting times for specific surgical procedures.

Our results are in line with previously published studies; in 2004 Minai et al. [11]. described little influence on perioperative management after cardiac consultation. Katz et al. concluded in two studies that consultation gave little advice that truly impacts management [10, 12]. An overuse of cardiac consultation was reported by Aslanger et al. in 2011 [13] after examining 712 referrals to the cardiologist. The only study with a conclusion in contrast to these findings is from Kleinman et al. [9]. in 1989, who described consultation as useful because it led to newly diagnosed hypertension and angina in 15% of their study group. Despite these new diagnoses there were no invasive interventions and in 72% of the cases no interventions at all were performed by the cardiologist. Furthermore, hypertension could easily be diagnosed during preoperative assessment without involving the cardiologist. All these outcomes are comparable with our findings, namely that a limited number of patients are being optimised during preoperative cardiology consultation.

The 2009 ESC guidelines for preoperative cardiac evaluation were created in order to help physicians select the best possible management strategies for the individual patient with cardiovascular disease [14]. This version of the guideline strongly emphasised starting perioperative β‑blocker therapy in high-risk cardiovascular patients, which is reflected in the reason for starting β‑blockers in our patient population. Surprisingly, β‑blocker therapy was initiated in nearly all the patients with a low risk for adverse cardiovascular events, reflected by a low Revised Cardiac Risk Index. Beneficial effects have been studied in patients with this low-risk profile but not proven and initiating β‑blockers is potentially harmful in perioperative management [20, 21]. A possible explanation for these prescriptions might be the cardiologist’s focus on long-term care instead of the specific preoperative work-up. Overall, the patients selected for preoperative cardiac consultation should be considered a low-risk population, given the incidence of 30-day mortality and MACE [7, 8].

Another aspect to be discussed is the potential problem that the physician initiating the consultation might not make it clear to the cardiologist why the consultation is being sought. This non-specific manner of referring may lead to a general diagnostic work-up and it is suggested as one of the causes of the little influence cardiology consultation has on perioperative management [5, 13]. The 2009 guidelines, as well as the current 2014 ESC and AHA guidelines, are not very specific about who should be referred for consultation ‘if the patient is unstable (unstable coronary syndromes, decompensated heart failure, severe arrhythmias, or symptomatic valvular disease), this condition should be clarified and treated appropriately prior to surgery’ [14, 18, 22]. This leaves a great open opportunity for sending patients for cardiology consultation.

Looking at our data, we feel that there is still room for a more efficient consultation. One option to better select patients suitable for cardiac risk modification would be to train anaesthesiologists to evaluate cardiac murmurs themselves, using transthoracic echocardiography in the outpatient clinic. Given the high percentage of consultations for cardiac murmurs, this could be a way to further improve efficiency in screening. Previous studies have shown that this might be a feasible option that can be further explored [23, 24].

Our study has several limitations that should be considered when interpreting our findings, such as its retrospective design, the lack of data concerning similar patients who were not referred and the lack of data concerning long-term mortality and morbidity. Secondly, the healthcare system in the Netherlands and the method of screening all preoperative patients at an outpatient anaesthesiology clinic might be different than elsewhere. Thirdly, due to the nature of our study, no firm conclusions can be drawn as to whether preoperative consultation should be omitted.

## Conclusion

In most cases, preoperative cardiac consultation does not change preoperative management, but could possibly delay surgery. A more careful consideration about the potential benefits of consulting must be made for every patient.
